# Long‐term monitoring of sporadic permafrost at the eastern margin of the European Alps (Hochreichart, Seckauer Tauern range, Austria)

**DOI:** 10.1002/ppp.2021

**Published:** 2019-09-15

**Authors:** Andreas Kellerer‐Pirklbauer

**Affiliations:** ^1^ Department of Geography and Regional Science, Working Group Alpine Landscape Dynamics (ALADYN) University of Graz Austria

**Keywords:** climate change, coarse debris layer, eastern European Alps, ground thermal regime, long‐term permafrost monitoring, rock glacier types

## Abstract

Delineating the spatial extent and the altitudinal lower limit of mountain permafrost is difficult due to complex topo‐climatic and variable ground thermal conditions within short distances. Little information exists regarding sporadic permafrost existence, its thermal characteristics and its long‐term changes at the eastern margin of the European Alps. To reduce this gap, permafrost monitoring was initiated in 2004 in the Seckauer Tauern mountains, Austria. Research was carried out in the summit region of Mt Hochreichart (2416 m a.s.l.) and at several nearby cirques and valleys, all with rock glaciers. Geomorphic mapping, numerical permafrost modeling, measurements of the bottom temperature of the winter snow cover, continuous ground temperature monitoring, electrical resistivity tomography and optical snow cover monitoring were applied. Results indicate sporadic permafrost occurrence in the summit region with mean annual ground temperatures slightly below 0°C at the surface and −1.4°C at 2.5 m depth. Permafrost lenses also exist in the transition zone between the rock glacier and the talus slope behind attributed to coarse‐grained, blocky material causing additional ground cooling. Thanks to long‐term data, statistically significant trends of atmospheric and ground warming were observed in 2000–2018. Permafrost at this site will presumably disappear within the next few decades.

## 1 INTRODUCTION

Knowledge of the spatial distribution of permafrost in the Eastern European Alps is based primarily on numerical models.^1^, ^2^ Such models have in common that they are built upon rather few directly measured point data both in time (short time series) and in space (limited number of field sites) and hence do not reproduce the spatial variability of permafrost at small scale. New classification algorithms can help to improve modeling performances (e.g. 3). Rock glacier and permafrost evidence inventories (e.g. 4) are often used as an important source for spatial permafrost modeling. However, it is not always straightforward to differentiate, for instance, between an intact and a relict rock glacier (e.g. 5) or to distinguish a rock glacier from a heavily debris‐covered glacier (e.g. 6, 7) because of its mixed ice‐debris nature evolving over a long period of time.^8^ Therefore, spatial permafrost models commonly have high uncertainties.

An alpine‐wide homogenous permafrost modeling approach is the Alpine Permafrost Index Map or APIM.^2^ According to the APIM, the extent of permafrost in Austria is between 484 and 2,907 km^2^ (0.6–3.5% of the national territory) depending on the permafrost index chosen as a threshold. A reasonable index of ≥0.5 yields a permafrost area of 1,557 km^2^, which is 25% of the modeled permafrost in the entire European Alps. The index describes semi‐quantitatively the occurrence of permafrost, from permafrost in nearly all conditions to permafrost only in very favorable conditions. The denomination highlights the problem that despite the fact that APIM is sophisticated in terms of the modeling approach, the environmental heterogeneities of the relevant mountain area cause substantial uncertainty in the model. Earlier estimates of permafrost distribution in Austria^1^, ^9^ have suggested that some 1,600–2,000 km^2^ of the country (1.9–2.4%) is underlain by permafrost. The field data used for these two national permafrost estimations were also very limited in time and space with vast areas without any permafrost‐related data.

**Figure 1:**
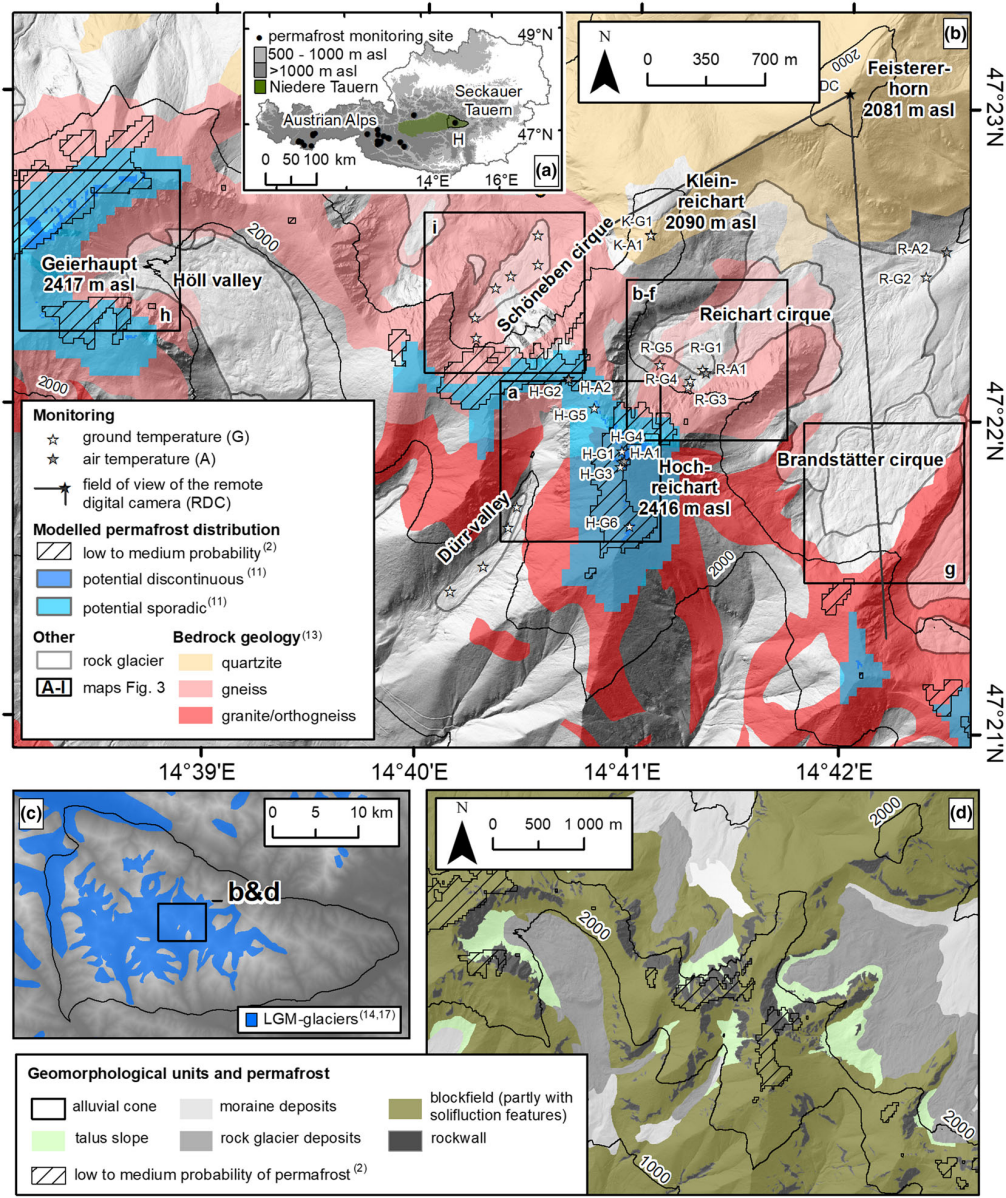
Map of the study area. (a) Overview map with location of the Hochreichart area (H) in Austria. (b) Location of the relevant subareas shown in Figure 3, modeled permafrost distribution,^2^, ^11^ extent of rock glaciers, bedrock geology (simplified from ref.^12^) and different automatic monitoring sites (for explanation of codes see Table 2). (c) Extent of glaciers during the Last Glacial Maximum in the Seckauer Tauern (based on refs 13, 14). (d) Geomorphological overview map of the Hochreichart area with modeled permafrost extent [Colour figure can be viewed at http://wileyonlinelibrary.com]

Monitoring of permafrost in Austria is carried out at some 23 study areas by different institutions.^10^ Most of the sites cluster around two regions in central and western Austria (Figure 1a). Knowledge of mountain permafrost existence and thermal conditions east of those sites and therefore also in the very east of the European Alps, a high‐alpine area still influenced by permafrost according to models (e.g. 2), is very limited. This paper attempts to answer the research question regarding present thermal conditions in a marginally permafrost‐affected area at the eastern margin of the European Alps and their relationships to present climate change. To gather such knowledge, permafrost monitoring was initiated in the Hochreichart area, Seckauer Tauern Range, in 2004 and has continued since then. Only preliminary results have been published so far.^11^ A comprehensive presentation and discussion of the permafrost‐related research at this site for the period 2004–2018 is the content of this paper. No such long‐term permafrost‐related data series have been presented earlier from anywhere in the Eastern European Alps apart from one study focusing on potential weathering of alpine rock walls.^15^

The aim of this paper is to present results on the detection, delineation, characterization and long‐term observation of a marginal permafrost site in the Eastern European Alps and discuss them in a broader spatial, topical and methodological context. The objectives are therefore to answer the following questions: (a) What is the spatial extent and the thermal characteristics of permafrost at the eastern‐most permafrost monitoring site of the European Alps? (b) How has permafrost changed since the beginning of the monitoring program in 2004? (3) How is this change related to climatic conditions? (4) What are the methodological problems and potentials in marginal permafrost detection and delineation?

## 2 STUDY AREA

The study area is located in the central part of the Seckauer Tauern mountains at 14°41′E, 47°22′N, extending 5.5 km from east to west and, respectively, 2.5 km from north to south. The Seckauer Tauern is a 626‐km^2^ large subunit of the Niedere Tauern, a mountain range located in central Austria. The main divide of the Seckauer Tauern runs roughly in a NW–SE direction. The highest summits in the Seckauer Tauern are the two mountains Geierhaupt (2417 m a.s.l.) and Hochreichart (2416 m a.s.l). Both summits are located within the area of interest of this study (Figure 1b).

**Figure 2:**
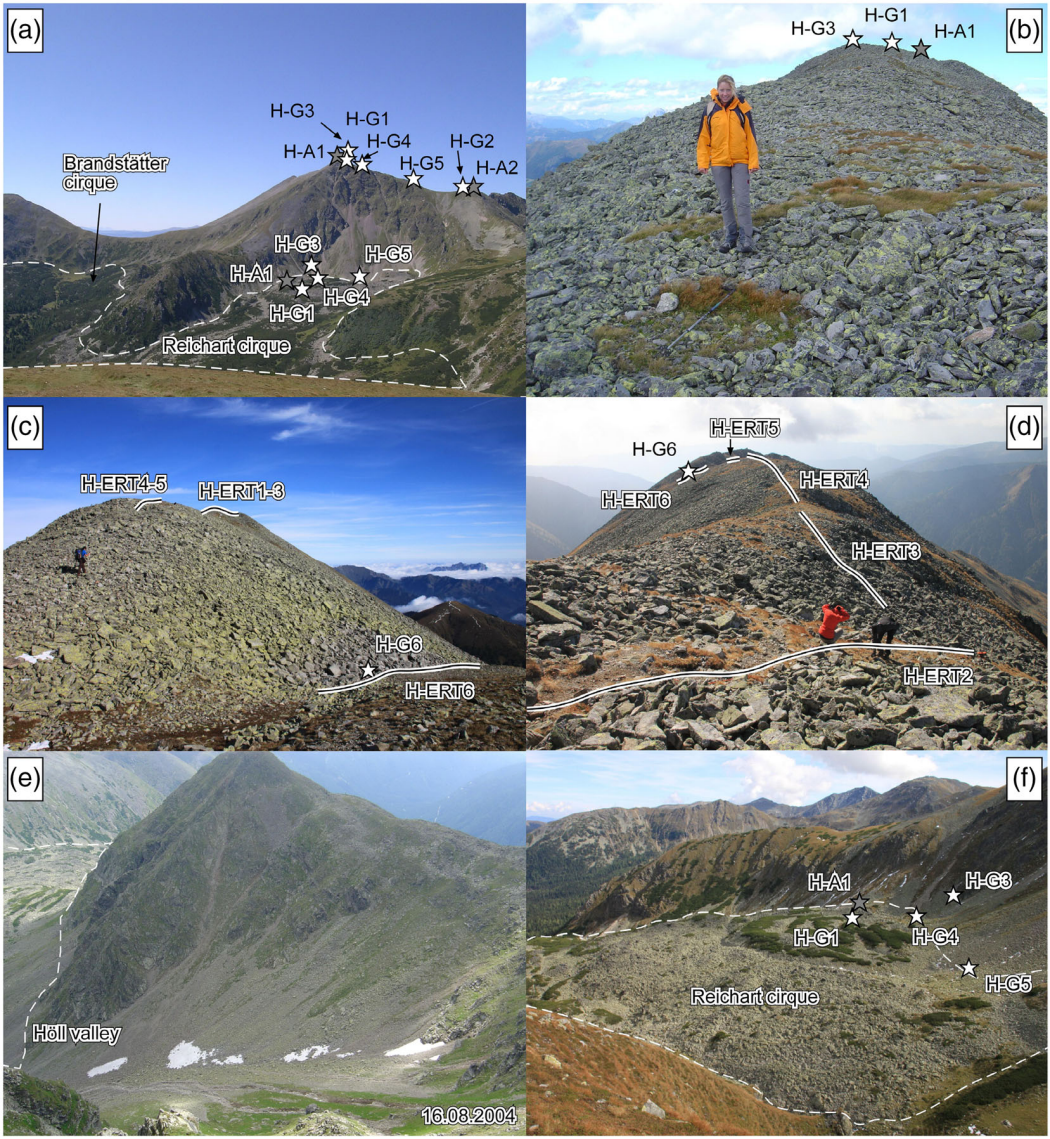
Terrestrial images of the study area Hochreichart: (a) Mt Hochreichart (2,416 m a.s.l.) and the Reichart cirque with the uppermost part of a polymorphic rock glacier as seen from the site of the remote digital camera (RDC); (b–d) summit area of Mt Hochreichart with coarse‐grained autochthonous blockfields with evidence of material sorting by frost action (note the vegetation patch with hiking stick in b); (e) Höll valley with late‐lying snow patches (cf. Figure 3h); (f) Reichart cirque with the upper part of a polymorphic rock glacier. Locations of relevant ground and temperature monitoring sites and profiles where geoelectrical measurements were carried out are indicated. Dashed line delineates rock glacier parts visible in the images. Photographs by a. Kellerer‐Pirklbauer [Colour figure can be viewed at http://wileyonlinelibrary.com]

Most permafrost‐related research in the area has focused on the summit region of the mountain Hochreichart and the neighboring Reichart cirque located north to north‐east of the summit. Coarse‐grained autochthonous blockfields, rectilinear slopes and solifluction landforms dominate the summit area and adjacent slopes of Hochreichart (Figures 1d and 2a‐d ). As judged from the widespread lichen coverage, the well‐developed weathering rinds and the general stable appearance of these sediments, it can be assumed that the present morphodynamical dynamics at these blockfields is insignificant. Bedrock uncovered by autochthonous block material is not very widespread. Such exceptions are steep cirque headwalls or the flanks of U‐shaped valleys (Figures 1d and 2a,e,f). The bedrock geology in the summit areas of the Seckauer Tauern is dominated by granites, orthogneisses and acid gneisses as well as quartzites and conglomerates to the north‐east of the main drainage divide^12^ (Figure 1b). Further areas of interest in this study were the Brandstätter and Schöneben cirques, the Höll and Dürr valleys, and the summit area of the Kleinreichart. Well‐developed rock glaciers characterize all the three cirques and the two valleys of interest.^13^, ^16^

The rock glaciers consist predominantly of coarse‐grained, blocky, gneissic sediments at the surface. Coarse‐grained talus and finer‐grained debris‐flow sediments occur at their routing zones and the talus slopes. The rock glaciers exhibit in general a rugged topography with bent transverse and longitudinal ridges and furrows. Such ridges are sometimes covered by dwarf pines (*Pinus mugo*) interspersed with Swiss stone pine (*Pinus cembra*), European larch (*Larix decidua*) or spruce (*Picea abies*) accentuating the flow structures (Figure 2f). Continuous woody vegetation partly exists at the lower part of the rock glaciers. The two large rock glaciers in the Höll valley and Reichart cirque are also some of the largest of the entire Eastern Alps.^17^ Rock glaciers in the Seckauer Tauern had started to form early in the Lateglacial period because of glacier‐hostile climatic conditions in the area even during the Last Glacial Maximum (LGM) around 20 k a BP^13^, ^14^ with only valley and cirque glaciers during that time (Figure 1c). Mean annual air temperature (MAAT) and mean annual precipitation (MAP) at an automatic weather station located in the study area at 1,512 m (site R‐A2 in Figure 1b) were, respectively, 3.9°C and 1,422 mm in 2000–2018. A theoretical zero‐degree isotherm at 2,100 m a.s.l. is calculated based on a lapse rate for the standard atmosphere of 0.0065°C m^–1^.

## 3 MATERIAL AND METHODS

### 3.1 Numerical permafrost modeling

The development of new permafrost models was not within the scope of this paper. Earlier modeling approaches are, however, relevant for the regionalization of the collected and interpreted data and hence are summarized here. Permafrost distribution was quantified using empirical and statistical models. First, the relationship between MAAT and permafrost was analyzed. Second, empirical values of probable lower limits of discontinuous permafrost occurrence of a nearby region in combination with local correction values were used. For details on these two approaches refer to Kellerer‐Pirklbauer.^11^ Third, results from an alpine‐wide homogenous statistical permafrost model based on alpine‐wide permafrost observations, MAAT, potential incoming solar radiation and precipitation^2^ are considered here.

### 3.2 Bottom temperature of the winter snow cover

The bottom temperature of the winter snow cover (BTS) is known to be an indicator for the presence or absence of permafrost in alpine terrain. Based on studies in the Western Alps, empirically established thresholds were defined: permafrost unlikely at >−2°C, permafrost possible at −2 to −3°C, and permafrost probable at <−3°C.^18^ Interpretation of the measured BTS values should be made very carefully. The two main assumptions are that BTS remains constant in mid‐winter (a winter equilibrium temperature [WEqT] exists) below a snow cover of >0.8 m and the BTS value is controlled by the heat flux from the subsurface.^19^

BTS was measured at 394 points, in four different cirques/valley heads and in late winters between 2004 and 2012 at snow cover thicknesses of at least 0.8 m during the time of measurement (Table 1). A PT100 thermocouple probe fixed to the bottom of a 3‐m‐long steel rod (System KRONEIS) was used. During the first BTS campaign on March 21, 2004 a single BTS value was based on only one‐point measurement. During the other eight campaigns a single BTS record was the averaged value of two to four measurements within a 1‐m^2^ area.^19^ BTS campaigns were carried out several times in the Reichart cirque to study interannual variations in the uppermost part of the rock glacier. BTS was not measured every year at exactly the same locations. The spatial autocorrelation of BTS records at distances less than 200 m is generally high although measurements within small distances might show considerable variability due to vegetation, soil, snow and measurement errors.^19^ The point data within a 0.04‐km^2^ area in the Reichart cirque were therefore considered as suitable for interpolation (inverse distance weighting) and subsequent statistical comparison. BTS values were tested regarding normality (Kolmogorov–Smirnov test) and correlated against elevation, snow depth and potential shortwave radiation during the generally snow‐free period (JJASON).

**Table 1:** Summary of the nine BTS campaigns accomplished between 2004 and 2012 in different cirques and valley heads of the study area: Reichart = Reichart cirque, Brandst. = Brandstätter cirque, Höll va. = Höll Valley, Schöneb. = Schöneben cirque. Each individual meausurement site is indicated in Figure 3

Site	Date	BTS‐sites	Elevation (m a.s.l.)			Temperature (°C)			Snow depth (cm)			Permafrost interpretation (*n*)		
		(*n*)	Min.	Max.	Median	Min.	Max.	Median	Min.	Max.	Median	No.	Pos.^a^	Prob.^b^
Reichart	21.03.2004	26	1,925	1,972	1,935	−5.1	−0.1	−2.1	100	270	185	11	7	8
Reichart	19.03.2005	26	1,813	1,966	1,928	−5.8	‐0.2	−1.8	110	275	198	13	7	6
Reichart	03.04.2007	78	1,803	1,987	1,925	−3.7	−0.1	−0.7	80	290	165	61	14	3
Reichart	05.04.2008	97	1,803	1,994	1,928	−4.1	−0.1	−2.0	85	285	150	47	31	19
Reichart	02.04.2009	70	1,811	1,987	1,927	−5.1	−0.1	−1.2	95	290	190	43	11	16
Brantst.	03.04.2009	35	1,756	2,031	1,883	−2.6	−0.1	−0.4	98	220	145	29	6	0
Höll va.	17.03.2007	30	2,037	2,211	2,135	−2.3	−0.1	−0.3	100	300	183	27	3	0
Schöneb.	13.03.2012	18	1,749	1,907	1,814	−5.9	−0.9	−3.5	115	230	175	5	3	10
Schöneb.	26.03.2012	14	1,864	1,923	1,882	−4.4	−0.2	−1.5	95	260	203	7	5	2

a Possible permafrost (−2 to −3°C).

b Probable permafrost (<−3°C).

### 3.3 Automatic monitoring

A network for continuous ground and air temperature monitoring by using miniature temperature data loggers in the Hochreichart area was initiated in October 2004 and steadily expanded. Over the years, ground temperature was measured at 12 sites: six at the Hochreichart summit area, five in the Reichart cirque area and one in the Kleinreichart summit area (Table 2, Figures 1 and 2). Air temperature was monitored at five sites in close spatial distance to ground temperature monitoring sites. The installed devices are one‐ or three‐channel data loggers (GeoPrecision) equipped with PT1000 temperature sensors measuring and logging hourly the temperature. According to the producer, the PT1000 temperature sensors have an accuracy of +/−0.05°C, a range of −40 to +100°C and a calibration drift of <0.01°C yr^–1^. At the air temperature monitoring sites (*n* = 5), the loggers are protected from direct insolation by radiation shields (Young). Sensors at 0 cm depth are shielded from direct solar radiation by thin platy rocks that still allow rather unhampered air circulation within the voids. Raw data were checked and short data errors were corrected or filled using linear interpolation between the neighboring data points.^20^ Furthermore, data from a nearby automatic weather station (R‐A2) operated since 2000 by the Hydrological Service of the Federal Province of Styria were used.

**Table 2:** Automatic monitoring sites for ground (G) and air (a) temperature with length of data series. Meaning of the first letter in the site description: H = Hochreichart summit area, R = Reichart cirque area, K = Kleinreichart summit area. For location see Figures 1, 2 and 3

Site	Elev. (m a.s.l.)	Aspect	Slope (°)	Morphology	Substrate	Sensors^a^ (cm)	Data series (MMYY‐MMYY)
H‐G1	2,416	NNW	25	Summit plateau	Coarse debris	0	1004–0818
H‐G2	2,285	NW	7	Ridge	Coarse debris	0	0815–0917
H‐G3	2,415	WSW	36	Edge of summit plateau	Void in an autochthonous blockfield	−250	0911–0818
H‐G4	2,393	WSW	24	Ridge	Coarse debris	0	1006–0808
H‐G5	2,303	NW	22	Ridge	Coarse debris	0	1006–0818
H‐G6	2,363	E	20	Small valley in debris	Coarse debris	0	1014–0818
H‐A1	2,412	NW	38	Summit plateau	Coarse debris	200	1007–0917
H‐A2	2,285	S	37	Ridge	Coarse debris	200	0815–0818
R‐G1	1,920	NW	14	Rock glacier surface	Coarse rock glacier	0, −50, −100, −125	1005–0818^b^
R‐G2	1,559	E	21	Edge of rock glacier	Coarse debris	0, −70	0615–0618
R‐G3	1,960	N	75	Rock wall	Bedrock	−3, −10, −40	3 cm: 0806–0818 All: 0608–0818
R‐G4	1,935	NNE	29	Talus slope	Coarse debris	0	1004–1005
R‐G5	1,954	NE	30	Talus slope	Coarse debris	0	1004–1005
R‐A1	1,925	NNW	6	Rock glacier surface	Coarse debris	200	0808–0818
R‐A2^c^	1,512	E	3	Front of rock glacier	Fine‐grained material	200	1000–0307 and 0108–1118
K‐G1	2,090	NNE	9	Summit plateau	Coarse debris	0	0815–0818
K‐A1	2,091	E	11	Summit plateau	Coarse debris	200	0815–0818

a Sensors at 0 cm depth are shielded from direct solar radiation by thin platy rocks.

b Variable time series for the different sensor depths.

c R‐A2 is operated by the Hydrological Service of the Federal Province of Styria, data kindly provided.

Mean annual data series were computed for monitoring years (August 1 to July 31) to account for the normal fieldwork month August where temperature data are commonly retrieved from the loggers. Ground and air temperature have also been monitored since 2011 at the Dürr valley and the Schöneben cirque, respectively (Figure 1, white stars, 10 monitoring sites in the two valleys). Temperature monitoring at these two areas focuses on hydrological issues, although the cooling effect of the blocky layer is also considered.^21^, ^22^ Relevant thermal results from these two areas are considered in the discussion.

For each temperature monitoring site, mean annual ground (MAGT) or air (MAAT) temperature, seasonal snow cover days (SCD) and winter equilibrium temperature (WEqT) were calculated. SCD was estimated by calculating the sum of days with a considerable snow cover damping effect. SCD values were considered as such when the weekly standard deviation of the mean daily ground surface temperature was ≤0.25°C.^23^ WEqT is the (rather) stable mean temperature in February and March and was calculated for sites with a SCD of at least 3 months indicating (widely) decoupled air and ground temperatures during winter. In more detail, the calculated WEqT value was the mean value of the two monthly values for February and March. Furthermore, the surface offset was calculated for neighboring air and ground surface temperature stations, i.e.namely H‐G1/A1, H‐G2/A2, R‐G1/A1, R‐G2/A2, R‐G2/A2 and K‐G1/A1. The annual surface offset, which is the difference in temperature between the air and the ground surface,^24^ was calculated for five sites with neighboring air and ground temperature monitoring sites.

An automatic remote digital camera (RDC) is located near the summit of the Feistererhorn (Figures 1b and 2a) taking daily pictures from the Hochreichart mountain and the Reichart Cirque. The RDC consists of a timer control unit (DigiSnap 2000), a digital camera (Nikon Coolpix 5400), and a weatherproof case all placed on top of a 1‐m‐high pile of rocks. The camera took daily images at 10 a.m. for different periods (altogether 995 days) between November 26, 2006 and November 9, 2012. The pictures have been used here only to assess visually the snow cover conditions (e.g. extent of snow cover, blown‐off areas) and dynamics (e.g. snow fall event/extent) in the Reichart cirque and the summit area.

### 3.4 Electrical resistivity tomography

Electrical resistivity is a physical parameter, which is related to the chemical composition of a material and its porosity, temperature, and water and ice content.^25^ Two‐dimensional electrical resistivity tomography (ERT) uses multi‐electrode systems and two‐dimensional data inversion in order to receive a rather accurate model of the subsurface.^26^ ERT was applied at altogether 24 profiles at different places in the study area. Profiles H‐ERT1 to H‐ERT6 were carried out in the summit plateau of Hochreichart in 2014. Profiles R‐ERT1 to R‐ERT7 were measured in the Reichart cirque in the transition zone of a talus slope and a rock glacier in 2015 and 2016. R‐ERT8 was also measured at the same location but in 2008. Results of R‐ERT8 have been published.^27^, ^28^ Profiles D‐ERT1 to D‐ERT3 were accomplished in the rooting zone depression and the upper end of the rock glacier in the Dürr valley. Profiles S‐ERT1 to S‐ERT7 were all measured in the talus slopes and the rooting zone of the rock glacier in the Schöneben cirque (Table 3).

**Table 3:** Overview of ERT profiles measured in the study area. For location see Figures 2 and 3. Meaning of the first letter of the ERT code: H = Hochreichart summit area, R = Reichart cirque area, D = Dürr valley, S = Schöneben cirque. *In Figure 10

Code of ERT profile	Date	Elevation (m a.s.l.)		Electrodes		Profile length (m)	Annotation
		Lower	Upper	Spacing (m)	Number (*n*)		
H‐ERT1*	07.10.2014	2,406.0	2,411.8	2	25	48	Parallel to H‐ERT2
H‐ERT2*	07.10.2014	2,410.7	2,411.0	2	25	48	Parallel to H‐ERT1
H‐ERT3	07.10.2014	2,399.7	2,406.8	2	25	48	Slightly overlapping with H‐ERT4
H‐ERT4*	07.10.2014	2,400.0	2,400.8	2	25	48	Slightly overlapping with H‐ERT3
H‐ERT5	07.10.2014	2,395.3	2,396.4	2	25	48	South of H‐ERT4
H‐ERT6*	07.10.2014	2,356.6	2,367.5	2	25	48	Near an almost perennial snow patch
R‐ERT1*	17.06.2015	1,919.7	1,985.8	4	45	176	Intersecting with R‐ERT4 at 50 m
R‐ERT2*	17.06.2015	1,919.7	1,943.75	2	50	98	Intersecting with R‐ERT4 at 50 m, lower part of R‐ERT1
R‐ERT3*	17.06.2015	1,925.8	1,948.9	4	21	80	Intersecting with R‐ERT4 at 24 m
R‐ERT4*	17.06.2015	1,923.3	1,943.8	2	50	98	Intersecting with R‐ERT1/2 at 18 m and with R‐ERT3 at 24 m
R‐ERT5	16.06.2016	1,919.7	1,928.5	2	25	48	Lower part of R‐ERT1; intersecting with R‐ERT7 at 34 m
R‐ERT6	16.06.2016	1,925.8	1,933.3	2	25	48	Lower part of R‐ERT3; intersecting with R‐ERT7 at 18 m
R‐ERT7	16.06.2016	1,923.3	1,928.0	2	25	48	Lower part of R‐ERT4; intersecting with R‐ERT5 at 20 m and with R‐ERT6 at 33 m
R‐ERT8	24.08.2008	1,919.1	1,964.0	2.5	50	120	Course in Figure 3 of Niesner et al. (2010)
D‐ERT1	18.06.2015	2,001.0	2,004.6	2	25	48	Parallel to a late lying snow patch
D‐ERT2	18.06.2015	1,993.7	2,003.1	4	22	84	Intersecting with D‐ERT2 at 65 m
D‐ERT3*	18.06.2015	1,984.3	1,988.2	2	44	86	Intersecting with D‐ERT3 at 77 m
S‐ERT1*	26.06.2014	1,920.8	2,027.3	4	49	192	Profile along vegetation line
S‐ERT2	26.06.2014	1,944.9	1,994.3	2	41	80	Identical to 80–160 m of S‐ERT1
S‐ERT3*	14.10.2014	1,945.5	2,004.5	4	25	96	Lower end same as S‐ERT4; identical to 48–96 m of S‐ERT0
S‐ERT4	14.10.2014	1,945.5	1,973.5	2	25	48	Lower end same as S‐ERT3; identical to 48–96 m of S‐ERT
S‐ERT5	14.10.2014	1,915.0	1,941.3	2	25	48	Transition to rock glacier
S‐ERT6*	14.10.2014	1,820.7	1,924.3	4	50	196	Overlapping with S‐ERT7
S‐ERT7	14.10.2014	1,804.8	1,870.7	4	41	160	Overlapping with S‐ERT6, bent

In 2008, an LGM‐Lippmann 4‐Punkt light hp resistivity‐meter using the Wenner‐Alfa configuration was used. During all other campaigns a GeoTom‐2D system (Geolog2000) with multicore cables has been used applying both the Wenner and the Schlumberger arrays.^26^ Saltwater was sometimes used at the electrodes to improve electrical contact. ERT data analyses were carried out in RES2DINV concatenating both array results (in the case of good‐quality concatenated Wenner and Schlumberger data with a root mean square [RMS] error < 10) or by using only the Wenner data alone (in the case of poor quality Schlumberger data). The apparent resistivity data were inverted using robust inversion modeling. Bad datum points were removed before the inversion. The number of iterations was stopped when the change in the RMS error between two iterations was small.^29^

Granitic rocks are characterized by resistivity values mostly around 5 k Ohm.m.^30^ Gneiss is in the range of 0.1–1 kΩ.m.^26^ Resistivity values for frozen material can vary over a wide range from several kΩ.m to even millions of Ω.m (see^26^ and references therein). The resistivity value range 10–100 kΩ.m was considered as permafrost in the area of interest. The lower boundary is a rough estimate for permafrost existence. Values <10 kΩ.m were considered as bedrock and finer‐grained sediments with variable soil water content. The upper boundary follows previous suggestions^27^, ^28^ based on experiences at the rooting zone of a rock glacier at profile R‐ERT7. Higher values are regarded as coarse‐grained and mainly dry sediments with open voids and hence high porosity. However, permafrost cannot be excluded at values <10 and >100 kΩ.m.

## 4 RESULTS

### 4.1 Modeled spatial permafrost distribution

**Figure 3:**
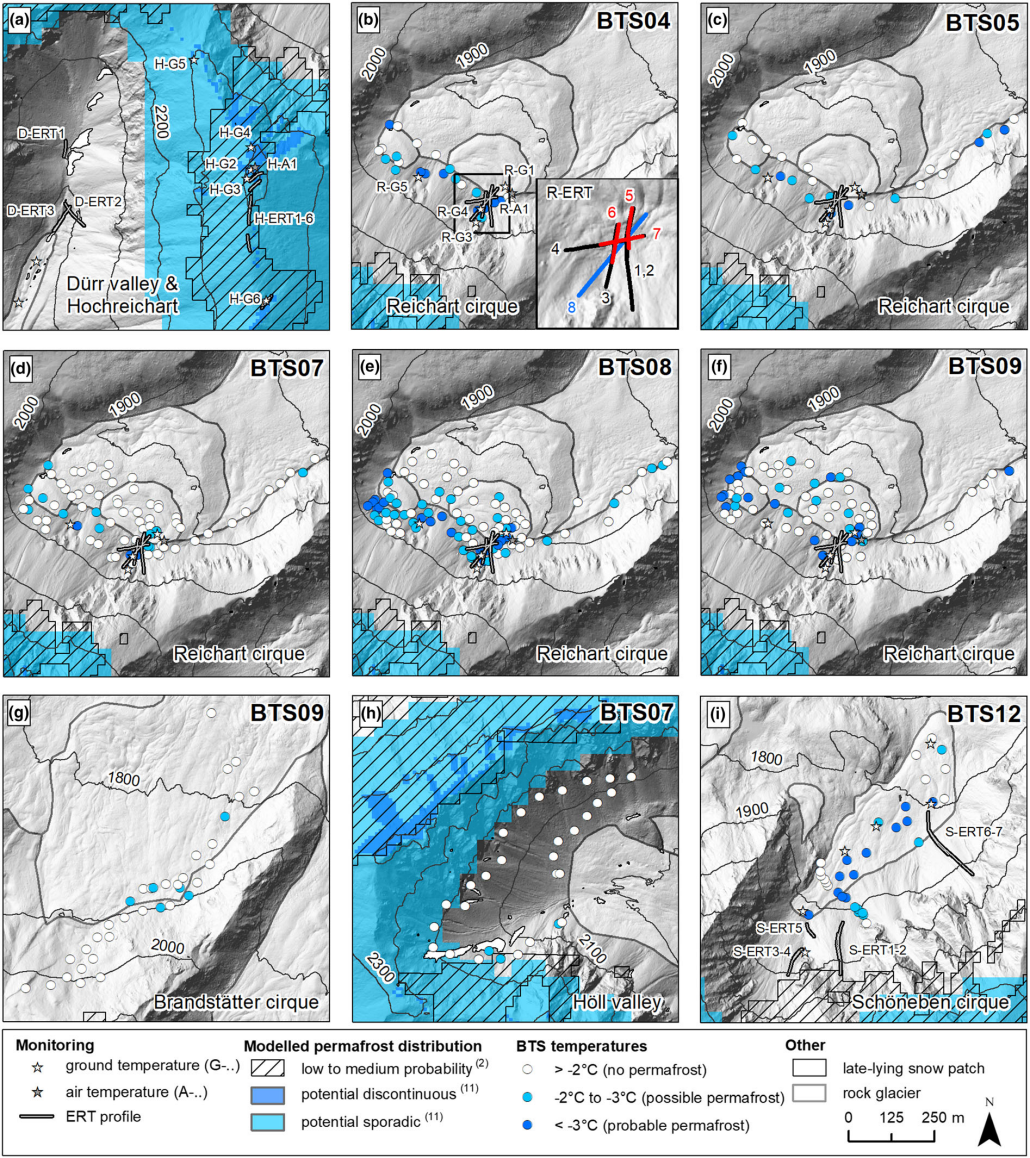
Maps of subareas of the Hochreichart area with location of monitoring sites (Tables 2), spatial extent of modeled permafrost distribution, late‐lying seasonal snow patches (July conditions), and results of nine different BTS campaigns between 2004 and 2012 (for details see Table 1). Modeled permafrost distribution are based on the literature^2^, ^11^ [Colour figure can be viewed at http://wileyonlinelibrary.com]

Permafrost distribution according to the APIM approach^2^ reveals low to medium probabilities of permafrost occurrence in the study area. The lowest area potentially underlain by permafrost according to this approach is about 2,000 m a.s.l. at a north‐facing slope in the Schöneben cirque. The lower limit of low‐to‐medium probable permafrost at north‐facing slopes was slightly higher at the Reichart cique (2,110 m a.s.l.) and the Geierhaupt area. On east‐ and south‐facing slopes this lower limit was about 2,400 m a.s.l., whereas on west‐facing slopes it was at about 2,300 m a.s.l. Permafrost distribution according to empirical relationships^11^ revealed potential sporadic permafrost for slightly larger areas compared to APIM with a mean lower limit of about 2,200 m a.s.l. Potential discontinuous permafrost is restricted to very small areas at highest elevations apart from north‐facing slopes, where discontinuous permafrost might exist as low as 2,270 m a.s.l. Importantly, all cirques with their rock glaciers were always modeled as permafrost free (Figures 1 and 3).

### 4.2 Winter temperature conditions based on BTS

**Figure 4:**
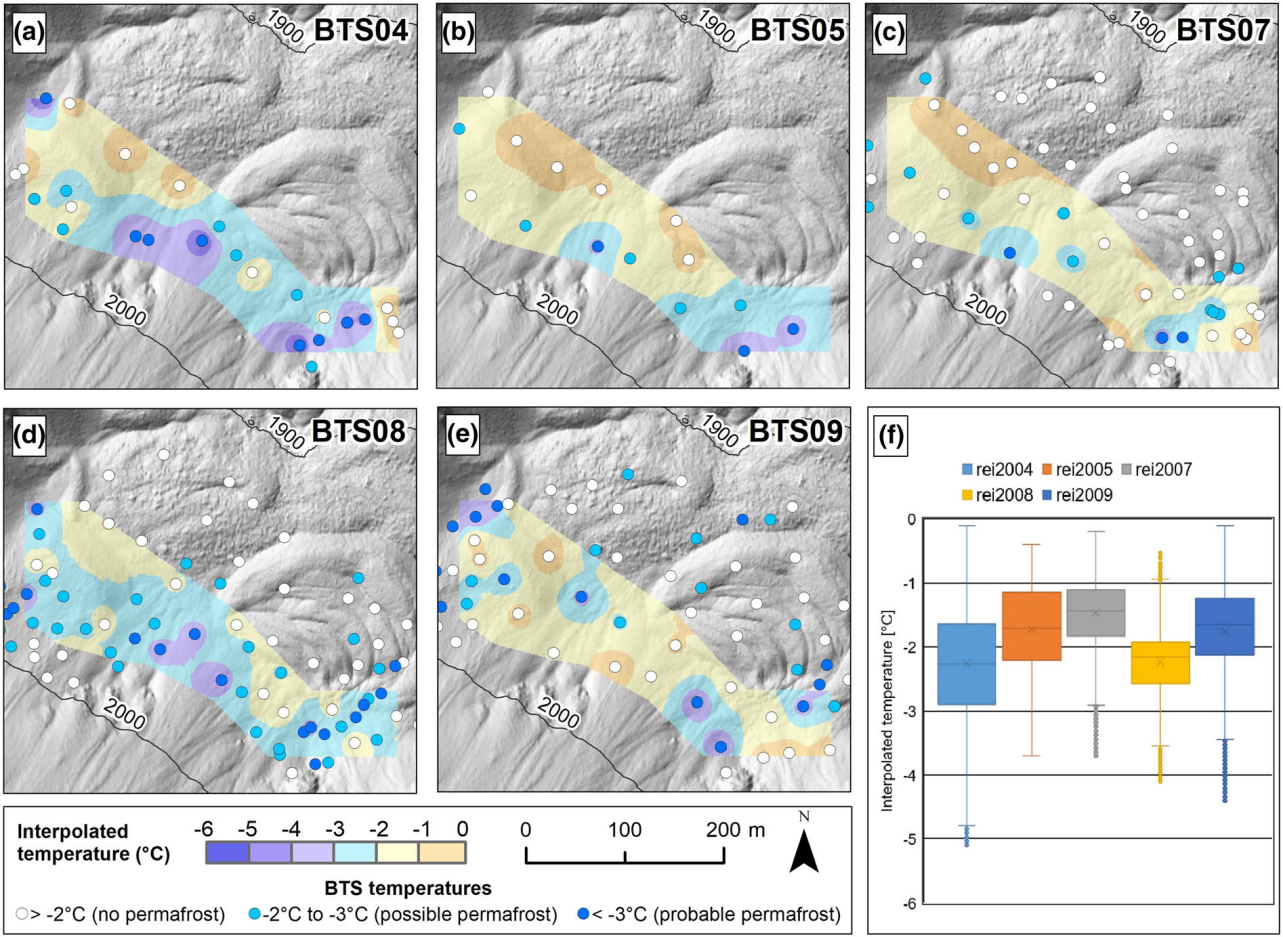
Reichart cirque: BTS measurements and interpolated temperatures in 2004, 2005, 2007, 2008 and 2009 (a–e) and summary box‐plot diagram for a 0.04‐km^2^ area in the rooting zone of a large polymorphic rock glacier for these five years (f) [Colour figure can be viewed at http://wileyonlinelibrary.com]

Table 1 summarizes the results from all nine BTS campaigns. Figure 3 depicts the location and results of all 394 BTS measurement sites in the different areas. Figure 4 shows measured and interpolated BTS results for a 0.04‐km^2^ area (i.e. the area with the interpolated values in Figure 4a‐e) in the Reichart cirque for the years 2004, 2005, 2007, 2008 and 2009. Table 4 lists the Pearson correlations between BTS‐temperatures versus potential short wave radiation (PSWR) during JJASON, elevation and snow depth. The measured BTS data varied between −5.9 and −0.1°C at snow depths of 0.8–3.0 m. In total, 61.8% of all BTS values indicate permafrost absence. Possible and probable permafrost are indicated at, respectively, 22.1 and 16.1% of all measurement sites. As judged from the BTS data, permafrost is very unlikely in the Höll valley (Figure 3h) and the Brandstätter cirque (Figure 3g), whereas it is quite widespread in the Reichart and Schöneben cirques (Figure 3b‐f, i). BTS results in the Reichart cirque from the different years show large interannual variability. However, in all years with BTS data the highest probability for permafrost existence seems to be in the uppermost part of the rock glacier and the adjacent talus slope as well as at the foot of a steep N‐ to NW‐facing rockface at elevations down to 1,850 m a.s.l.

**Table 4:** Pearson correlations between BTS temperatures and, respectively, PSWR during JJASON, elevation and snow depth. Bold character = correlation significant at the 0.01 level. Normal character = correlation significant at the 0.05 level

Site	Date	BTS vs.		
		PSWR	Elev.	Snow depth
Reichart	21.03.2004	–	–	–
Reichart	19.03.2005	**0.61**	–	–
Reichart	03.04.2007	**0.25**	–	–
Reichart	05.04.2008	**0.21**	**–0.29**	0.22
Reichart	02.04.2009	–	–	–0.29
Brantst.	03.04.2009	–	–	–
Höll va.	17.03.2007	0.66	0.45	–
Schöneb.	13.03.2012	–	**–0.75**	–
Schöneb.	26.03.2012	–	–	–0.64

Median values for the 0.04‐km^2^ area with interpolated BTS values range from −2.27°C (2004) to −1.44°C (2007), averaging −1.84°C. Correlation analyses between BTS‐temperatures and, respectively, PSWR during JJASON, elevation and snow depth revealed significant correlations for all but two BTS campaigns (Table 4). Results show that higher solar radiation values (PSWR) and lower elevations correspond to higher BTS‐temperatures. In the case of variable slope aspects, the latter relationship does not exist (i.e. Höll valley, Figure 3h). The relationship between snow depth and BTS‐temperature is less clear and varies between sites. Results indicate either the thicker the snow cover during the date of measurement the higher the BTS value (Reichart cirque in 2008) or vice versa, i.e. the thicker the snow cover the lower the temperature (Reichart cirque in 2009, Schöneben cirque on March 26, 2012).

### 4.3 Ground and air temperature

**Figure 5:**
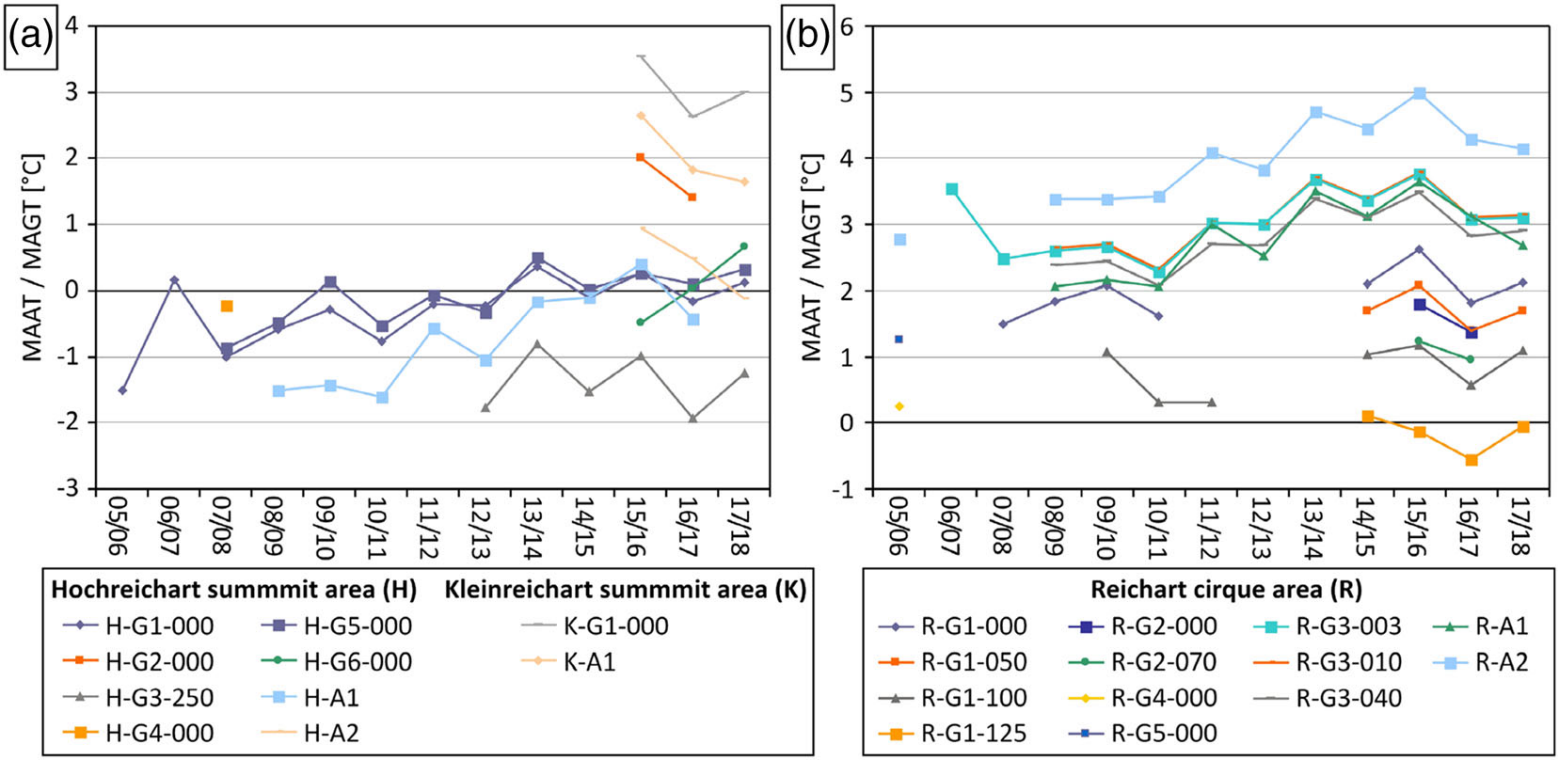
MAAT and MAGT at the surface (000) and at different depths (003–250; in cm) during the period 2005/06 to 2017/18 for all sites with respective data: (a) for the summit areas Hochreichart and Kleinreichat; (b) for the Reichart cirque. For location of sites refer to Figure 3 [Colour figure can be viewed at http://wileyonlinelibrary.com]

Figure 5 depicts MAAT and MAGT at the surface (000) and at different depths (between 3 and 250 cm) during the period 2005/06 to 2017/18. Note the general warming trend for some ground (H‐G1, H‐G5, R‐G1, R‐G3) and air temperature (H‐A1, R‐A1, R‐A2) monitoring sites with longer time series. This trend was quite steady until 2015/16 but has been more unclear since then. Mean annual values at the Kleinreichart summit area are always positive, while those at the ground surface at the Hochreichart summit vary around 0°C, suggesting marginal permafrost existence. The values at site H‐G3 at 2.5 m depths are, however, always clearly negative (range − 1.0 to −1.9), show no trend for the period 2012/13 to 2017/18 and are supportive for permafrost existence. In the Reichart cirque all but one sensor show positive mean annual temperatures. Only the sensor at 1.25 m depth of site R‐G1 had slightly negative mean values in three out of four years with appropriate data. In addition, at site R‐G4 the mean annual value at the surface (only one year with appropriate data) was merely slightly positive, suggesting at least some permafrost favorability at the southern margin of the rock glacier in the Reichart cirque.

**Figure 6:**
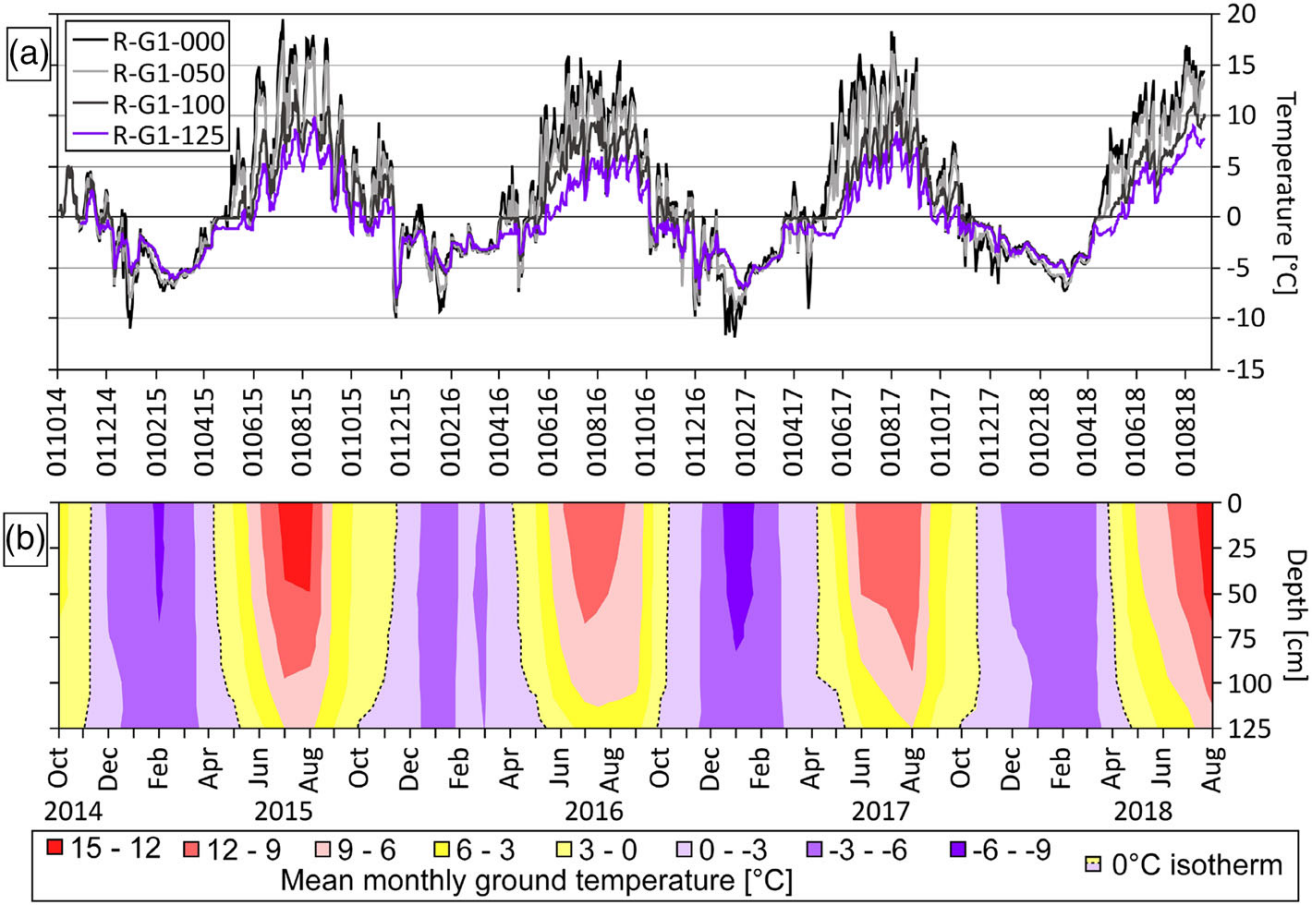
Ground thermal conditions at the coarse‐grained rock glacier site R‐G1 between October 2014 and August 2018. (a) Mean daily ground temperature at the surface and at three different depths. (b) Subsurface isotherms (3°C intervals) based on mean monthly values. The dashed line marks the 0°C isotherm [Colour figure can be viewed at http://wileyonlinelibrary.com]

**Figure 7:**
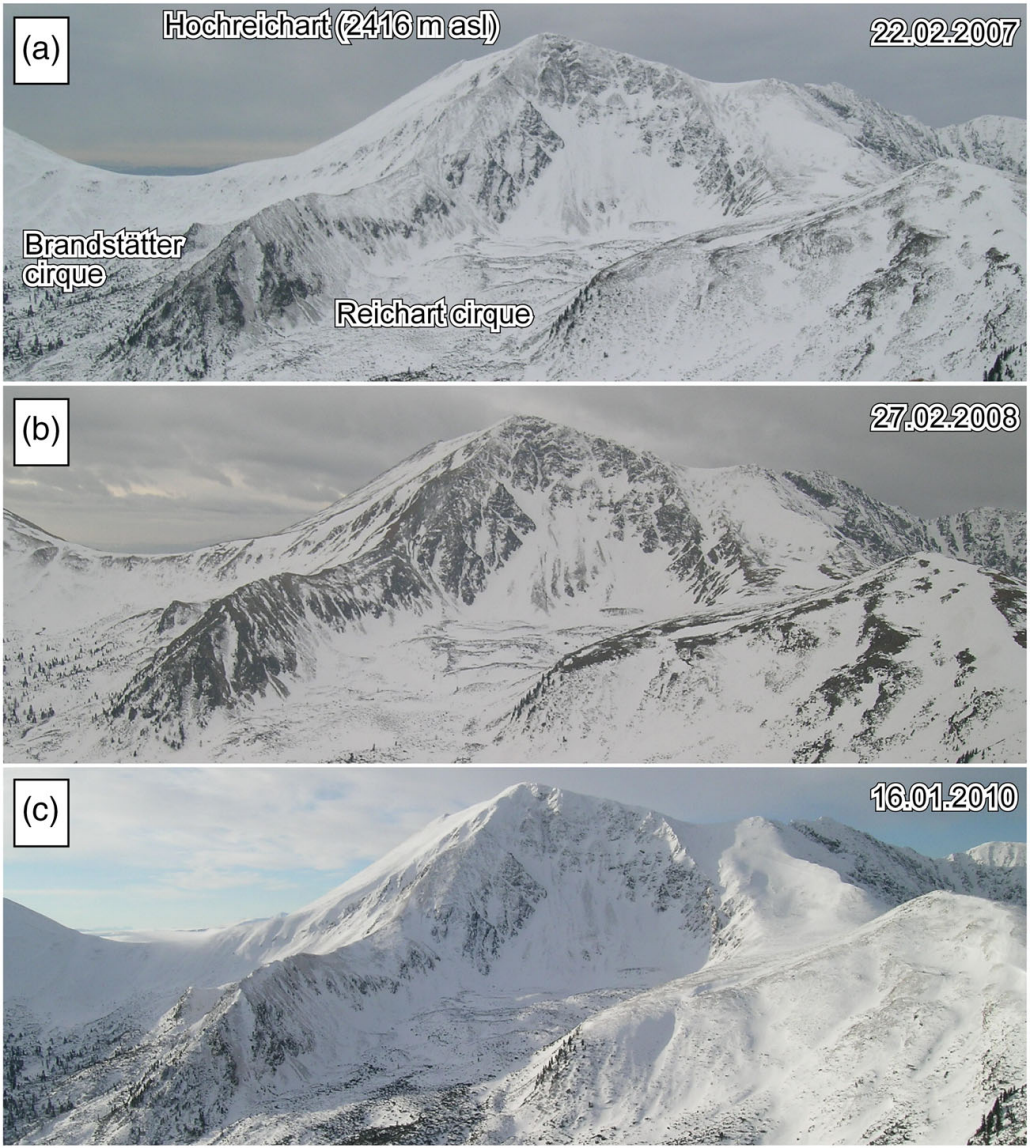
Pictures of the Reichart and (partly) Brandstätter cirques as seen from the automatic RDC in late winter (a and b) and mid‐winter (c) indicating typical snow cover conditions during those times. Only the relevant cutout of the RDC images is shown. Note the snow distribution pattern in the cirque, the talus slope behind and the rockwalls around [Colour figure can be viewed at http://wileyonlinelibrary.com]

Figure 6 depicts the mean daily temperature at four different depths and the corresponding subsurface isotherms for the coarse‐grained rock glacier site R‐G1 during the period October 2014 to August 2018. As indicated in this graph, there was no clear winter equilibrium temperature with stable thermal conditions in February and March in any of the four winters between 2014 and 2018. The calculated mean SCD at this site based on the surface data (R‐G1‐000) was only 26 days (range 14–48) during the four winters 2014–2018. The images from the Reichart cirque taken by the automatic RDC give an impression of the spatial pattern of the seasonal snow cover. Typically, in late winter (February–March) the transverse ridges and convex slopes in the rooting zone of the rock glacier are snow‐free or only sparsely covered by snow (Figure 7). Large single blocks or cairns penetrate through the winter snow cover allowing a better ground–atmosphere coupling. At site R‐G1 this coupling is very efficient with rather fast responses to atmospheric warming or cooling. The lowest sensor at site R‐G1 at a depth of −125 cm reveals positive mean monthly temperatures for only four months per year (normally June to September) and yields – as indicated above – a mean annual value which is commonly negative. The neighboring sensor (in terms of the vertical profile) at 100 cm depth (R‐G1‐100) already shows 6 months of positive temperatures (May–October).

**Figure 8:**
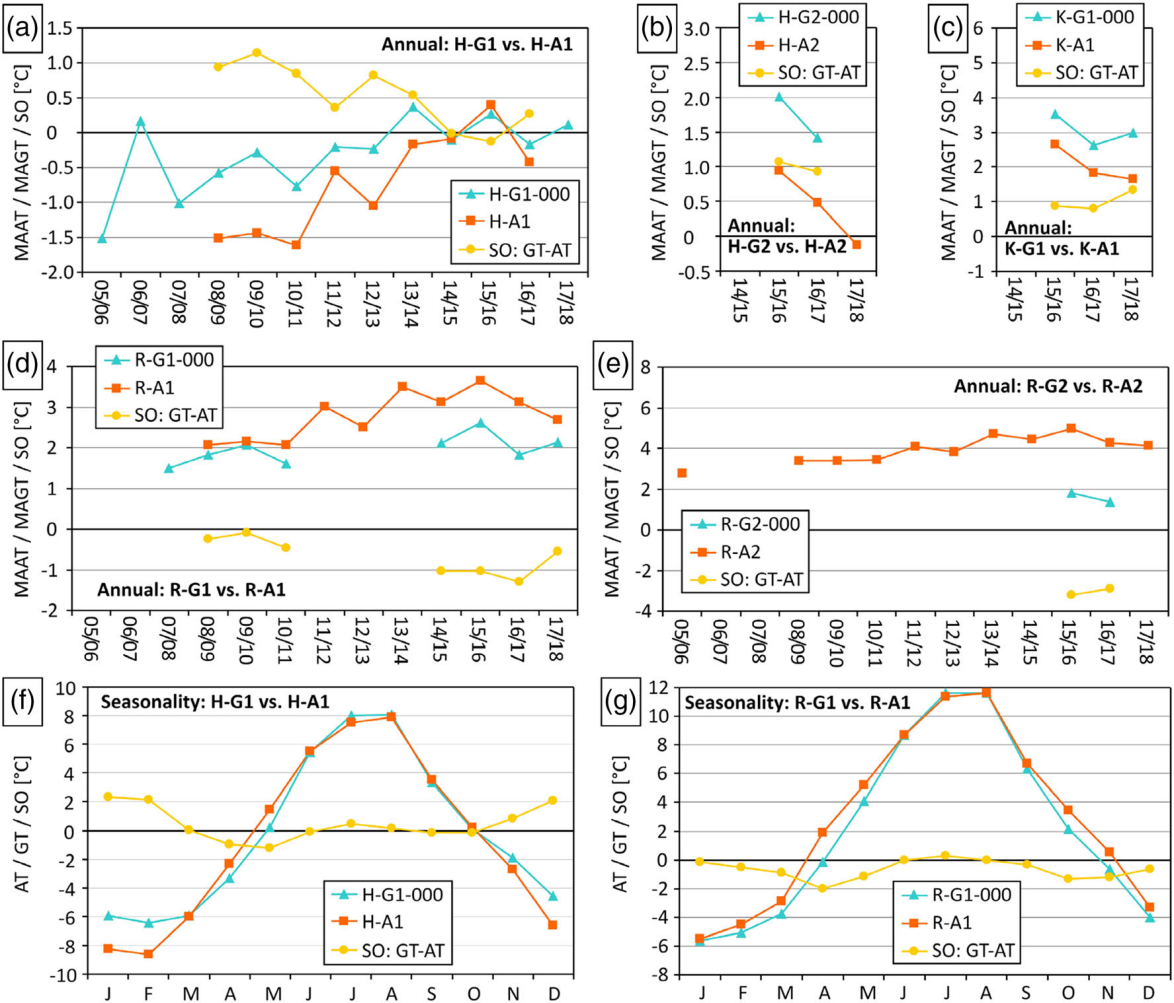
Surface offsets in the Hochreichart area based on adjacent ground surface and air temperature monitoring sites: comparison of annual air temperature, ground surface temperature and resulting surface offset values for the summit areas of Hochreichart (a, b) and Kleinreichart (c), Reichart cirque (d) and the frontal area of the rock glacier which originates in the Reichart cirque (e); comparison of mean monthly air temperature, ground temperature and resulting surface offset values for the summit area of Hochreichart (f) and the Reichart cirque (g) [Colour figure can be viewed at http://wileyonlinelibrary.com]

The annual surface offset values at all three data logger pairs in summit positions (Figure 8a–c) are in all but one case (*n* = 14) positive, indicating higher ground surface temperatures compared to the air temperature at *c*. 2 m height. The mean annual surface offsets at the summit stations are 0.5°C for the pair H‐G1/H‐A1 and, respectively, 1.0°C for the two pairs H‐G2/H‐A2 and K‐G1/K‐A1. However, the surface offset values for the two data logger pairs at the Reichart rock glacier are strikingly different. At the upper part of the rock glacier, the comparison of air and ground surface temperature yield for all seven years with respective data negative surface offsets in the range −0.1 to −1.3°C (Figure 8d). At the rock glacier front the surface offset yields substantially lower values with −3.2°C for 2015/16 and −2.9°C for the 2016/17 (Figure 8e). This very high surface offset for an alpine environment is primarily related to the relatively low ground temperature monitoring site (R‐G2) where cold air effusion is sensible even during warm periods in spring and summer. It must also be kept in mind that R‐G2 is at 47 m higher elevation (horizontal distance 180 m) compared to R‐A2.

For the two logger‐pairs with longer data series, the seasonal variation of the surface offset is plotted in Figure 8(f,g). At both pairs a similar wave pattern was observed with relatively high values in winter and summer and lower values in spring and autumn. However, in more detail the picture is as follows. At the rock glacier site (Figure 8g) the surface offset is negative in all but one month (July), which means for almost all months lower temperatures at the ground surface compared to the air. This pattern is substantially different at the Hochreichart summit area (Figure 8f) with seven months with positive surface offsets and only five months with negative ones.

**Figure 9:**
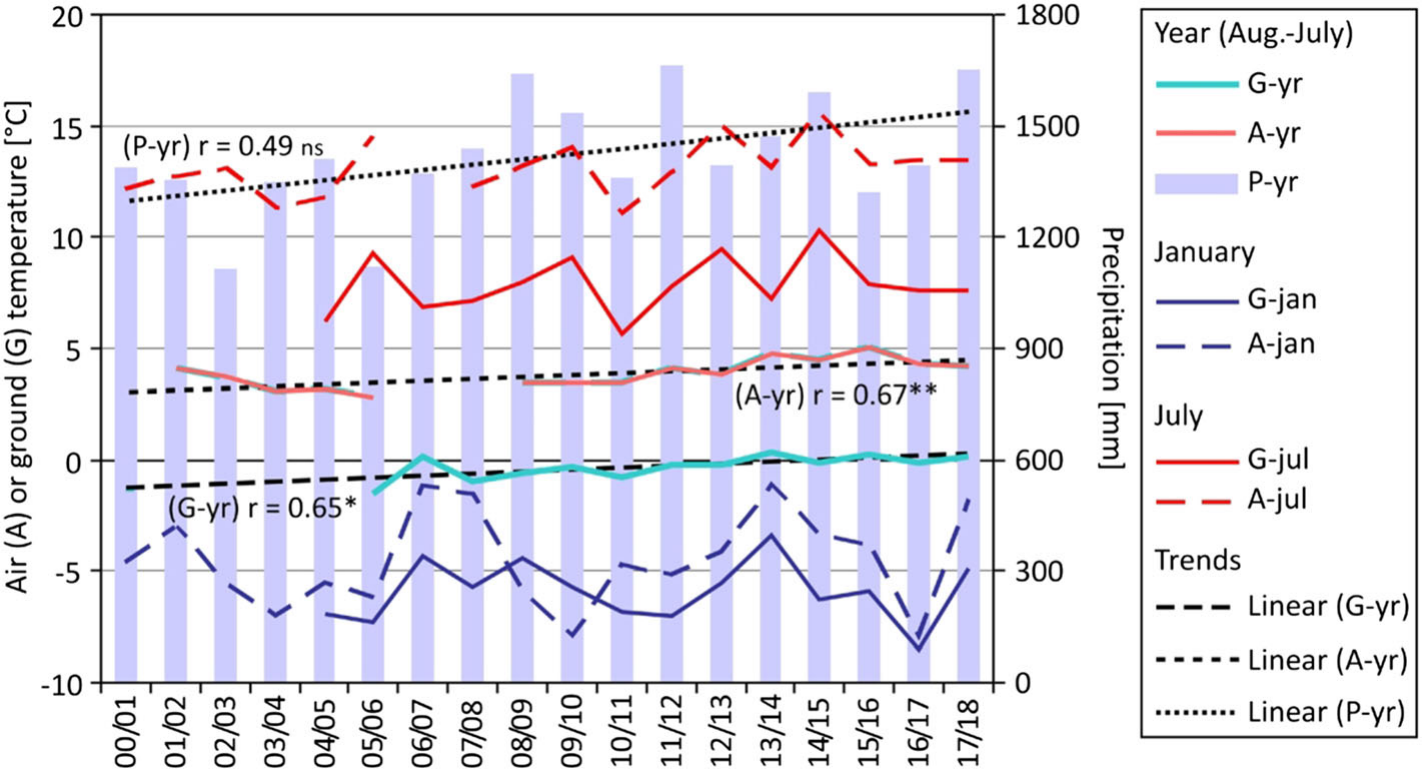
Evolution of ground temperature (H‐G1 at 2,416 m a.s.l.; G), air temperature and precipitation (both R‐A2 at 1,512 m a.s.l.; A and P) between 2000 and 2018 in the Hochreichart area for annual and selected monthly (January, June) time series. Linear trends for annual means/sum are indicated. Significance: **P < 0.01, *P < 0.05, ns = not significant [Colour figure can be viewed at http://wileyonlinelibrary.com]

Figure 9 depicts the evolution of the mean annual values for ground surface temperature (at H‐G1), air temperature and precipitation (both at R‐A2) for the period 2000–2018 (with gaps). The mean monthly temperature values for January and July are additionally shown. The linear trends for annual data indicate a rather strong and statistically significant increase in the mean annual air and ground temperatures as well as a tendency of increasing annual precipitation values. Such trends are less clear for monthly data. Whereas the July values are characterized by a warming trend, although with large interannual variations, there is no recognizable trend for the January data. The correlation between ground and air temperature is significant in all cases: strong for annual (*r* = 0.90) and in particular for July (*r* = 0.97) and still moderate for January (*r* = 0.66). The last is related to the influence of seasonal snow cover.

### 4.4 Subsurface conditions based on geophysics

**Figure 10:**
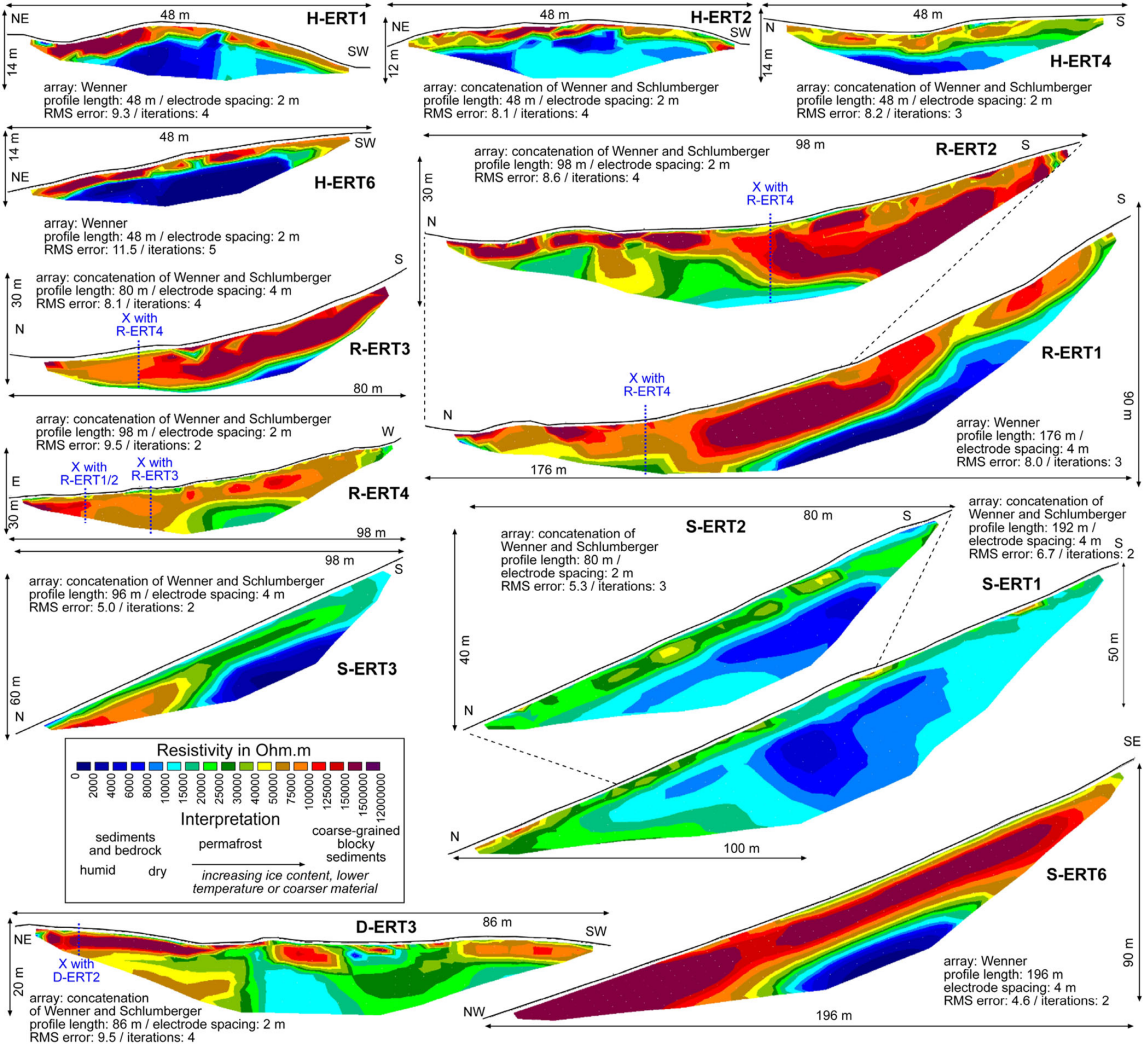
Electrical resistivity tomography inversion results for 13 profiles measured in the study area. For technical details refer to Table 3. Locations of profiles are shown in Figure 3. “X” indicates the location where two ERT profiles intersect. Note that the spatial scale varies between profiles [Colour figure can be viewed at http://wileyonlinelibrary.com]

Results from 13 representative (out of 24) ERT profiles are depicted in Figure 10. Four profiles are shown for each of the three areas: Hochreichart summit area, Reichart cirque and Schöneben cirque. The results from a 86‐m‐long profile (D‐ERT3) measured in the Dürr valley are additionally presented. A clear layer structure with high to very high resistivity values in a several‐meter‐thick top layer (about 1–4 m) and low to medium high resistivity values below this layer was revealed for all profiles in the Hochreichart summit area. Most of the very high values in the surface layer can possibly be attributed to open voids in the autochthonous blocks. However, several zones in the upper layer along the profiles do not necessarily exclude permafrost existence below an active layer of several meters. The lower layer is presumably bedrock (orthogneiss). Values exceeding 10 kΩ.m observed in the lower of the two layers, particularly of H‐ERT 1 and H‐ERT2, might indicate deeper permafrost.

Results from the four depicted ERT profiles in the Reichart cirque (R‐ERT1–4) show a substantially different pattern. In these profiles up to three distinct layers can be identified particularly at the profiles with 2‐m‐spacing resolution. R‐ERT1 is characterized by a downward‐thickening, 10–20‐m‐thick double‐layer of high to very high resistivity values covering bedrock. R‐ERT2 is part of the R‐ERT1 profile but in higher spatial resolution revealing an up to 5‐m‐thick layer with high to very high resistivities considered as a coarse debris layer with large open voids acting (partly) as the active layer. Below this top layer, a second layer with partly very high resistivities is evident. The shape of the elongated, very high resistivity area in R‐ERT1 and 2 suggests a large permafrost lens in the transition zone between the talus slope and the rock glacier. R‐ERT3, which runs parallel to R‐ERT1 and 2, shows a very similar pattern. Profile R‐ERT4 runs perpendicular to the other profiles and confirms the previous findings with a middle layer of high to very high values.

The first two profiles in the Schöneben cirque (S‐ERT1, 2) reveal a 5–7‐m‐thick upper layer interpreted as sediments partly with open voids covering bedrock. Both layers might contain permafrost, as indicated by the lenses in the upper layer and the higher values particularly at the lower end of the profile in the lower layer. S‐ERT3 reveals a similar pattern with *c*. 10 m of sediments covering bedrock, at least at the upper two‐thirds of the profile. The upper layer is again characterized by high values 3–5 m below the surface suggesting permafrost lenses. S‐ERT6 is very different in its pattern with an up to 2‐m‐thick upper layer (not occurring at the lower end of the profile) covering a very high resistivity layer of 20–25 m thickness (coarse grained, air‐filled, possibly permafrost) and bedrock. Finally, the depicted profile in the Dürr valley (D‐ERT) also shows layers with a *c*. 4‐m–thick upper layer with variable resistivities including some distinct lenses. The lower layer is much more diverse compared to the other profiles with high values even at 25 m depth from the surface, suggesting thick layers with air‐filled voids or even permafrost.

## 5 DISCUSSION

### 5.1 Spatial extent of permafrost: Modeling results versus temperature data

The existence of sporadic permafrost is strongly evidenced in the Hochreichart area by numerical modeling results, BTS data, ERT measurements and continuous ground temperature data. Continuous ground temperature monitoring revealed slightly negative MAGT at the summit plateau at 2,400 m a.s.l. (H‐G1, H‐G4). This is in rather good agreement with the altitude of the 0°C air temperature isotherm at 2,400–2,500 m a.s.l. in the Southern Eastern Alps^31^ suggesting the existence of active protalus ramparts^32^ in an even slightly warmer environment compared to the Hochreichart area at the same elevation. Similar mean ground surface values were measured at a north‐oriented site at about 2,300 m a.s.l. (H‐G5). Results from other slope orientations at similar elevations (H‐G2, H‐G6) indicate slightly warmer conditions supporting earlier modeling results.^2^, ^11^ Continuous ground temperature measurements in an open void at the summit plateau and about 2.5 m below the surface (H‐G3) revealed for a 6‐year period with mean annual temperature of −1.4°C with on average 8 months of negative temperatures. The horizontal distance between the two sites H‐G1 and H‐G3 is only 15 m. Hence, a mean annual thermal offset^24^ of at least −1.4°C can be assumed. An even higher thermal offset was revealed for the Reichart cirque at site R‐G1 (1920 m a.s.l.) with MAGT differences between the sensors at 0 and 125 cm depth of 2.0–2.7°C. Similar measurements at the rock glaciers in the Schöneben cirque and Dürr valley reveal comparable thermal offsets in the order of −0.5 to −1.8°C.^22^ Given the widespread existence of autochthonous blockfields, talus slopes and rock glacier deposits in the Hochreichart area as indicated in the geomorphological map, the measured low ground temperatures, and a thermal offset in such areas in the order of one to several degrees Celsius, one can assume permafrost conditions at many locations not considered by the spatial permafrost models.^2^, ^11^ This highlights the relevance of substrate and landform effects on permafrost distribution.^3^ However, a clear definition of a lower limit of permafrost in the study area is not trivial due to the site‐specific effects on ground thermal conditions just below or just above perennial freezing.

In addition, large blocks or piles of rocks forming natural cairns may penetrate the general terrain surface and the seasonal snow cover,^33^ allowing convective air flow into the coarse‐grained sediment layer.^34^ Such blocks, often darker in visual appearance due to the weathering rind indicative of minor snow protection during winter, exist in all the studied cirques. Warm air outflow (with hoarfrost crystals) from a hole in the snow nestled within such a large block was observed for example in the Reichart cirque in 2014, indicating convective heat transport in the open voids (cf. 21, 35).

### 5.2 Subsurface pattern of permafrost: Geophysical evidence

Results from the ERT measurements support the existence of sporadic permafrost not only in the summit area of Hochreichart but also in the cirques and valley heads nearby. Clear layer structures were revealed in all cases with lenses of high to very high resistivities often several meters below the surface. ERT data suggest frozen bedrock in the summit area of Hochreichart covered by a primarily not perennially frozen autochthonous blockfield layer. ERT results from the cirques indicate permafrost lenses at several profiles either in the talus slopes (Schöneben cirque) or sediment accumulations (Dürr valley) above the rock glaciers or in the transition zone to the rock glacier (Reichart cirque). Results from profile R‐ERT8^27^, ^28^ for this rock glacier ERT measurement site suggest permafrost at resistivity values of 50–100 kΩ.m. Along the profile, the results revealed an active layer of 2–4 m with two distinct thicker lenses at the rock glacier/talus slope transition zone (permafrost body >10 m) and at the lower part of the talus slope (permafrost body 5–6 m) and permafrost absence at the uppermost part of the profile. Similar results have been found based on ERT profiles in this study for the Reichart cirque (R‐ERT1,2,3) and partly for the Schöneben cirque regarding permafrost lenses at the talus slopes.

**Figure 11:**
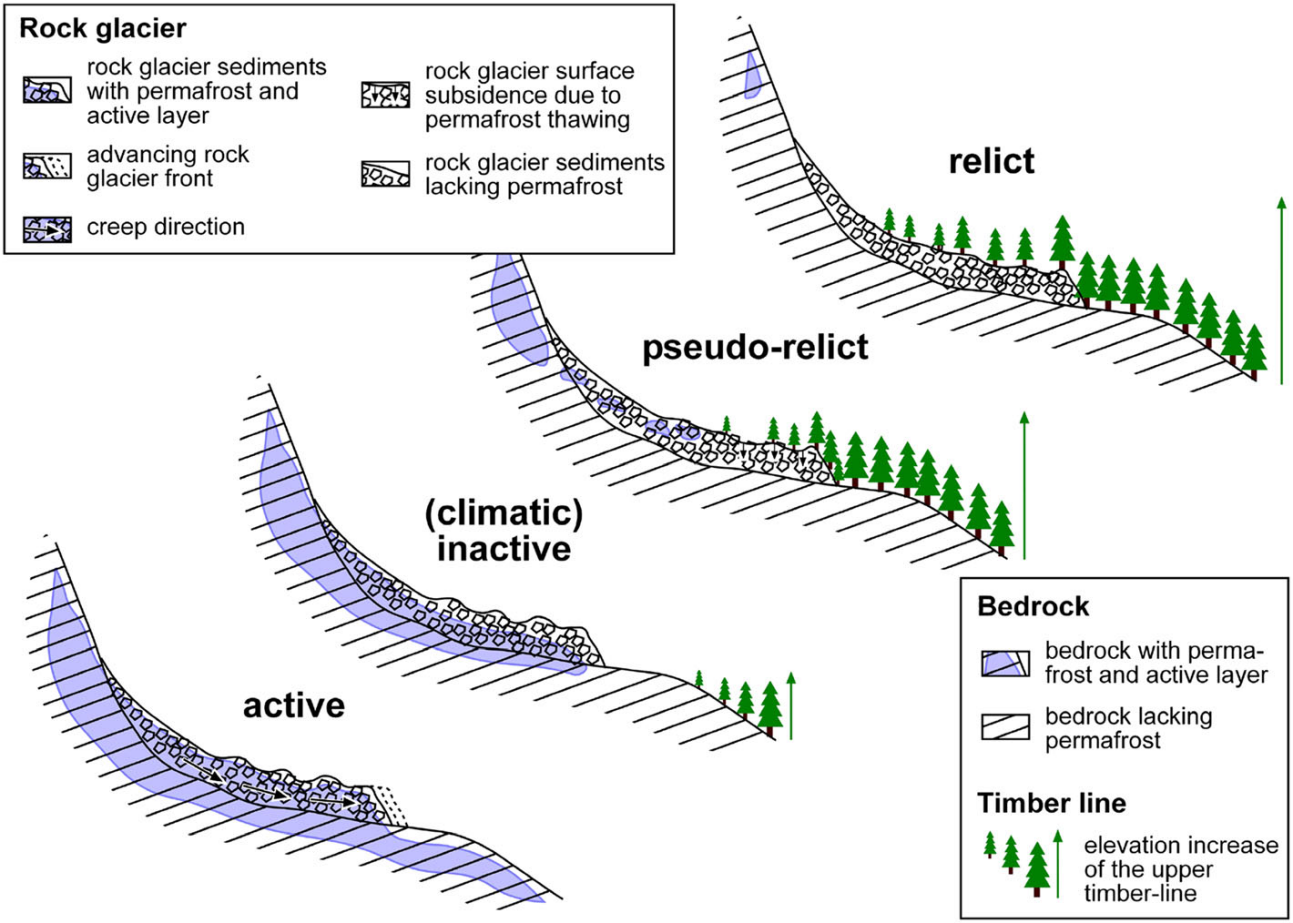
Rock glacier types as proposed previously.^37^, ^38^ The rock glacier in the Hochreichart cirque is considered to be pseudo‐relict, and hence a rock glacier which appears to be visually relict but still contains patches of permafrost (modified after 38). Changes of the timber line are a proxy for changes in vegetation [Colour figure can be viewed at http://wileyonlinelibrary.com]

Seismic refraction measurements were accomplished previously at three profiles for the central and lower part of the rock glacier in the Schöneben cirque.^36^ Results from this geophysical technique do not confirm permafrost at the rock glacier in the Schöneben cirque itself with maximum sediment p‐wave velocities of 1700 m s^–1^.^37^ No seismic refraction has been measured so far at the talus slopes behind but would be desirable in the future. The nature of overlapping resistivity values for different materials and the influence of air in voids causing higher resistivity values makes the interpretation difficult.^26^ Despite this restriction, it is evident that the results from the continuous temperature monitoring reveal suitable thermal conditions for permafrost not only at the summit area but also in the higher elevated parts of the cirques including the rock glaciers. Therefore, the rock glacier in the Reichart cirque is neither relict nor climatically inactive.^37^ It appears visually relict with collapse features at the lower part caused by ground subsidence processes and some vegetation cover, but contains lenses of permafrost at the upper end of the landform (Figure 11).

### 5.3 BTS data: What are they telling us?

Results from the BTS data measured in four cirques suggest marginal permafrost conditions with a dominance of “no permafrost” sites in all four cirques. In detail, interpretation of the BTS data should be made very carefully. For instance, the multi‐annual measurements in the Reichart cirque revealed substantial interannual variations although the general pattern of cooler and warmer areas in a given area remains very similar. Such interannual variations were detected also at other alpine areas (e.g. 39). This highlights the problem that using BTS data from only a single year for spatial permafrost modeling is substantially misleading. The spatial interpolation of BTS data for a 0.04‐km^2^ area for five different years (Figure 4) illustrates this problem with a moderate difference between the warmest and coldest year of 0.8°C. Permafrost assessments based on only one BTS campaign from one area must therefore be considered as lacking confidence.

BTS conditions in late winter are influenced by atmospheric temperature in early winter or in periods of thin snow coverage where cold air might penetrate through a thin or non‐existent snow cover. Hence, even if a snow cover thickness of >80 cm is measured during a BTS campaign,^18^ it necessarily gives little information about the existence or absence of a winter equilibrium temperature at the base of the snow cover. The BTS results presented in Figure 3(i) from the Schöneben cirque suggest probable permafrost for a rather large part of the rock glacier. However, as shown by continuous ground temperature measurements at this rock glacier (stars in Figure 3i; relevant data series shown in Figure 7c of ref. 21), the ground thermal conditions on the dates of measurements were not in equilibrium. A very cold period of up to −20°C in the first half of February 2012 severely and enduringly affected the thermal regime of all five ground temperature measurement sites (and hence the entire rock glacier). Therefore, the necessary assumption of a constant WEqT to apply BTS successfully was not fulfilled during the time of measurement. Hence, the expressiveness of the BTS data at least for the Schöneben cirque for 2012 is extremely questionable. This highlights the problem that if the snow cover history is not known at a BTS site, its expressiveness is again only limited. Only continuous ground surface temperature measurements over the entire winter allow the quantification and interpretation of the thermal regime at the snow–ground interface.^40^

### 5.4 Permafrost–climate relationships

Thanks to long‐term data gathered at this site, significant changes not only for mean annual air temperature (period 2000–2018) but also for ground temperature (2004–2018) were detected. A tendency of increasing precipitation since 2000 was revealed, although interannual variability was much higher. Ground and air temperatures in July have a strong correlation (*r* = 0.98). In contrast, the correlation of January ground to air temperatures is only moderate, attributed to variable nival influences common in alpine terrains (e.g. .^15^). Whereas a warming trend can be seen in July ground and air temperatures, no trend exists for January temperatures since the turn of the millennia. MAGT at the surface in the study area at the highest and coolest areas is only slightly below 0°C and hence a steady warming, which can be expected in the future,^41^ will lead to further ground surface warming and even to positive MAGT values at the coolest sites of the study area. Related to the efficient ground cooling due to the widespread existence of autochthonous blockfields, talus slopes and rock glacier sediments, and hence a general efficient thermal offset in the area causing lower temperatures at the top of the permafrost compared to the surface,^24^ permafrost will only slowly disappear from the Hochreichart area. A comparison of neighboring ground surface and air temperature conditions at five paired monitoring sites revealed positive surface offsets for the summit areas (i.e. higher ground compared to air temperature as expected; cf. 24), but negative surface offsets for rock glacier sites. The latter is attributed to efficient ground cooling of the coarse‐grained rock glacier sediments with a strong convective heat transport component.^42^, ^43^, ^44^, ^45^, ^46^ This surface offset is of the order of −0.7°C at the rooting zone of the Reichart rock glacier. At a location where deep circulating air within the rock glacier sediments emerges at the surface (similar observations were made at the Schöneben cirque^21^), the surface offset is even higher (−3.1°C).

On a seasonal scale, the offset varies between sites with thin block layers from sites with thicker rock glacier sediments. At the rock glacier site, the surface offset is negative in almost all months, meaning lower temperatures at the ground surface compared to the air. This indicates even in winter good thermal coupling of the (upper) rock glacier sediment layer with the atmosphere, a fact related to natural cairns and blocks as well as partly to thin snow coverage as discussed above.^33^, ^34^ Normally, the nival offset in winter is larger than the vegetation offset in summer,^47^ and hence MAAT is commonly lower than MAGT at the surface. Positive surface offsets on a seasonal scale are attributed to solar radiation heating the blocky surface during summer and to shielding effects of the seasonal snow cover against low temperatures during winter. Negative surface offsets around spring are related to snow cover insulation against high air temperatures and melting effects of the snow cover. Negative surface offsets in autumn are related to efficient ground cooling. The efficient coupling of air and ground temperature in winter helps to cause generally colder conditions at the coarse‐grained blocky surfaces compared to the surrounding (cf.^42^).

## 6 CONCLUSIONS

The combination of different methods revealed the existence of permafrost in the Hochreichart area. The widespread existence of autochthonous blockfields and coarse‐grained solifluction forms covering the bedrock is supportive for large thermal offsets and hence permafrost conditions at MAGT even close to 0°C. A clear delineation of permafrost is, however, not feasible due to the high porosity of the top layer. The pattern of high‐resistivity bodies, often occurring as distinct lenses, supports the existence of permafrost at talus slopes and the root zone of at least one of the rock glaciers in the area. Hence, the Hochreichart area is, to date, the most easterly evidence of existing permafrost found in the entire European Alps. Further to the east, maximum summit elevations are much lower and barely exceed 2,000 m. Lower elevations but also different dominant lithologies (limestone, dolomite) at higher elevations further delimit the spatial extent of coarse‐grained sediments favorable for permafrost conditions.

The long‐term monitoring of ground temperature data revealed a warming trend and confirms the ongoing atmospheric warming in the area. Whereas MAGT steadily increased in the period 2004–2018, the monthly values for summer (July) and winter (January) were far more variable. The warming trend is steered by warmer summers, whereas the winter temperatures show no trend.

This study also revealed some methodological drawbacks. ERT data are not straightforward to analyze and interpret, in particular when the substrate is rich in open voids with pore air. However, the pattern of high resistivity values in terms of distinct lenses below a surface (active?) layer often supports the assumption of permafrost. Furthermore, BTS data must be treated very cautiously in the case of a missing winter equilibrium temperature at the base of the snow cover. The uneven surface of bouldery rock glaciers favors atmosphere–ground air coupling even in mid‐winter. Furthermore, very cold periods might influence the ground surface even below thicker snow cover. Finally, multi‐annual BTS campaigns in the same cirque revealed substantial interannual differences. This implies that spatial permafrost models based on single BTS campaigns in a given area are highly vulnerable to fail because of some randomness in the data.
